# Novel insights into the geochemical evaluation of polymer drive composition on surfactant retention in carbonates using the surface complexation modeling

**DOI:** 10.1038/s41598-022-22422-7

**Published:** 2022-10-20

**Authors:** Ilyas Khurshid, Imran Afgan

**Affiliations:** 1 Khalifa University of Science and Technology, P.O. Box 127788, Abu Dhabi, UAE; 2 The University of Manchester, Manchester, M13 PL UK

**Keywords:** Crude oil, Engineering, Chemistry, Energy

## Abstract

Surfactant-polymer (SP) flooding is considered an efficient technique to increase the recovery of oil, especially from carbonates reservoirs, because of their complex nature. The objective of this study is to analyze the effect of polymer drive composition on surfactant retention. We developed a geochemical model that uses various surface complexation reactions at the mineral/brine interface, oil/brine interface, surfactant/brine interface, and oil/surfactant interface. We also incorporated four new surface complexation reactions that honor oil/surfactant geochemical interaction to determine the influence of polymer composition on surface retention for the first time. Then we validated the developed geochemical model against coreflooding experimental data. Additionally, we investigated the influence of various parameters of polymer drive on surface retention under high temperature and salinity using the suggested surface complexation model. The findings showed that our surface complexation model can estimate surfactant retention and its concentration in the effluent with a certain accuracy during polymer drive. The developed geochemical model is validated against single-phase and two-phase coreflooding experimental data. The findings revealed that for a more representative and accurate estimation of surfactant retention in chemical flooding, it is important to consider the oil/surfactant surface complexation reactions. Moreover, the detailed and comprehensive analysis showed that with the increase in temperature of the polymer drive, the retention of surfactant increases, and its concentration in the effluent decreases. The latter shows that surfactant retention is a more chemical process as opposed to physio-retention. It is also shown that the injection of a specific composition of polymer drive after a surfactant slug could decrease the surfactant retention, which is related to the force of repulsion between the ionic species and the rock surface. Moreover, the effect of hard ions (calcium and magnesium) in polymer drive is significant where the increase in the concentration of hard ions increases the retention of surfactant. Furthermore, it is important to mention that the lowest level of surfactant retention was achieved through a certain composition of polymer drive, thus the polymer solution dilution is not an effective approach. This is the first study to test a novel formulation of surface complexation modeling that considers the oil/surfactant effect on surfactant retention corresponding to the composition of polymer drive. The suggested framework to determine surfactant retention is conducted for harsh reservoir conditions of temperature and salinity and suggests that the surface complexation reactions for all rock-forming minerals must be considered.

## Introduction

During chemical flooding, the surfactants are injected to reduce the oil/water interfacial tension (IFT) and/or modify rock wettability. The surfactant slug is followed by polymer drive to avoid the chase water-fingering phenomenon^1^. A surfactant/polymer chemical flooding operation cannot be considered as two independent mechanisms that occur in a reservoir at the same time. The interaction of both chemicals affects the surfactant retention, operation economics, and oil recovery factor. In the polymer drive, the main ingredient of the drive solution is water and it comes from natural sources such as seawater or an aquifer, and in some cases, the produced water is also re-injected. In surfactant-polymer (SP) and alkaline-surfactant-polymer (ASP) flooding, the water is first treated to remove: impurities, suspended particles, and organic and inorganic particles. Then a certain amount of surfactants and polymers are added to the surfactant slug and polymer drive, respectively^2^ depending on the salinity of the injected. The concentration of surfactant and polymer is determined carefully because it controls the various geochemical processes and economics of the whole chemical enhanced oil recovery (EOR) operation.

**Figure 1:**
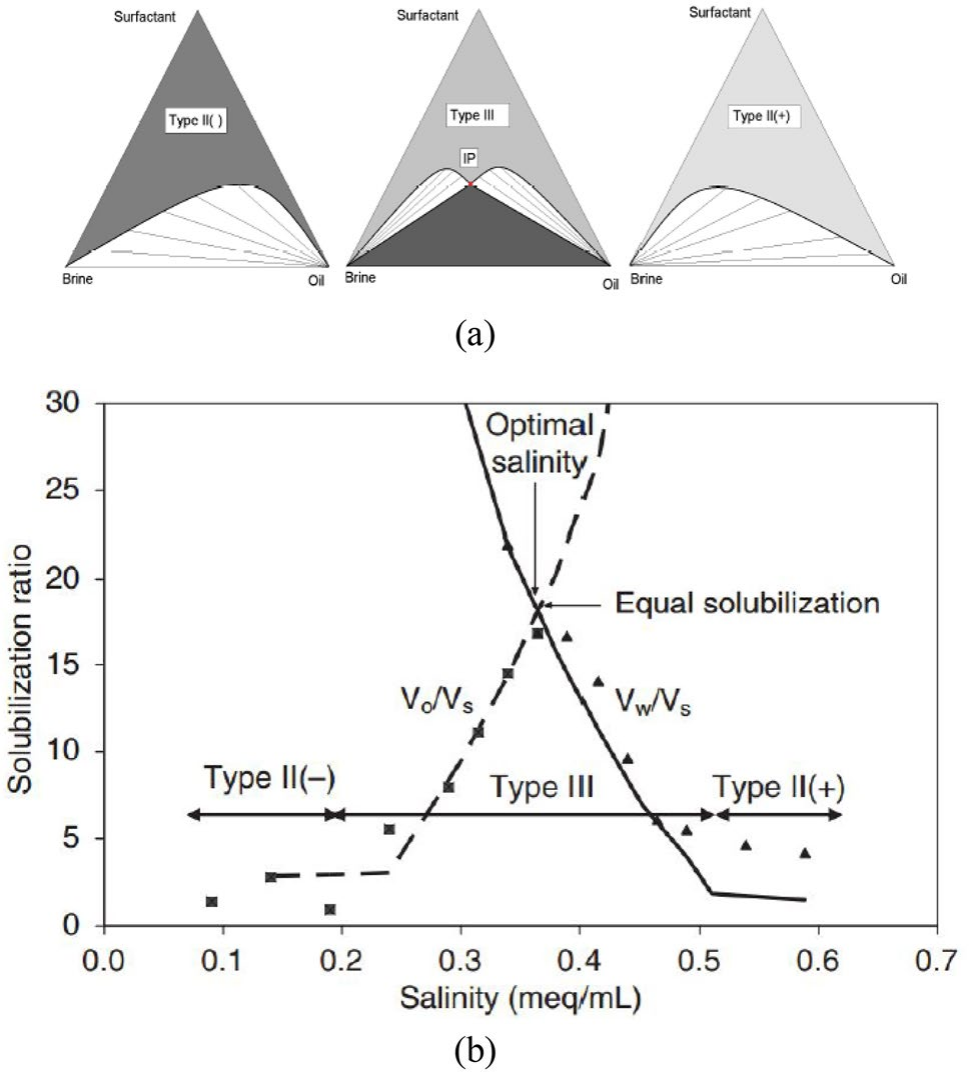
Types of microemulsions at different salinities (**a**) Microemulsion phase ternary diagrams at increasing salinity. (**b**) Solubilization ratio for oil and water for various salinities in a salinity scan^1^.

It is important to mention that the concentration of NaCl affects the activity and thereby the selection of surfactant during surfactant/polymer flooding. Since some surfactant fails to provide ultralow IFT values at low salinity conditions due to their chemical composition. However, these specific types of surfactant have the ability to improve the recovery of oil at high salinity conditions. On the other hand, the anionic surfactants are not desirable to use in high salinity conditions because they could precipitate, while interacting with NaCl ions^3^. Moreover, the NaCl ions would also reduce polymer viscosifying properties. Thus, in presence of high NaCl concentration, the use of anionic surfactant would form an inferior type of microemulsion leading to low oil recovery. The microemulsion is a thermodynamically stable liquid phase that is composed of surfactant, water, and oil. It originates in the reservoir when the water, oil, and surfactant mix with each other. Winsor^4^ characterized microemulsions into three types depending on their phase behavior. The phase behavior of a surfactant solution has distinct parameters and it is controlled by the composition and phase behavior of oil and brine present in the reservoir, the temperature, and pressure in a reservoir^2^. The microemulsions were classified by Winsor^4^ into three types. Type 1 is formed when the oil is in excess of water leading to the formation of the oil-in-water microemulsion. Type 2 originates when the concentration of the water phase increases and it attains equilibrium with the oil leading to water-in-oil microemulsion. Type 3 is different from both Type 1 and 2, it has excess saturation of both water and oil. Thus, it is a bi-continuous type of microemulsion. Type 3 is the most optimal interphase of microemulsion where the surfactant will successfully displace the adsorbed oil from the reservoir. Because for this type of microemulsion (type 3) achieves ultra-low IFT conditions, and high coalescence is attained by oil ganglia^5^. It is shown in Fig. 1 that an increase in salinity leads to the formation of Type 1 microemulsion that shifts to Type 3 and then to Type II^1^.

The polymers are used in chemical EOR floods to increase the viscosity, thus it is added to recover oil during chemical EOR operations. There are different types of polymers used to achieve this task. Among various types of polymers, hydrolyzed polyacrylamides (HPAMs) are the most widely used in the chemical EOR^6–8^. It is important to mention that the rheological properties HPAMs are controlled by the solution salinity based on their polyelectrolyte character^9,10^. Thus, the use of low saline solution due to polymer expansion because of electrostatic repulsion will increase viscosity^6^. Accordingly, decreasing the salinity of the flood would require several times lower concentrations of polymer that is needed in high salinity flood during chemical EOR, to achieve a similar target viscosity^11^. This technique assists in reducing the significant cost of polymer consumables. It is imperative to mention that this cost not only decreases the cost of various chemicals but also results in smaller injection facilities, logistics, and a low risk of the high concentration of back-produced polymer. Moreover, during the oil–water separation process, the treatment of produced water, and its disposal would become relatively less challenging and more cost-effective^12^.

During chemical EOR operations, when the concentration of surfactant is above critical micelle concentration (CMC), the formation of micelles will occur leading to a pronounced decrease in IFT. This reduction in IFT will displace the adsorbed oil ganglia from the pores. Thus, the oil ganglia coalesce and form an oil bank^1^. Moreover, the composition of injected polymer behind surfactant slug is important in maintaining mobility ratio and avoiding fingering phenomena during chemical flooding. Recently, it is suggested to optimize the injection rate and injection time of waterflooding and chemical flooding to improve the recovery of oil^13,14^ and minimize formation damage^15,16^. Thus, a better selection of surfactant and polymer, and optimizing solvent salinity will improve the fluid phase behavior. Thus, it is important to develop a combination of various chemicals during chemical EOR for a certain reservoir with distinct reservoir fluid composition and reservoir rock properties. Therefore, this technique will assist in developing an integrated approach to prepare a chemical recipe that will successfully decrease the residual oil saturation.

Moreover, during surfactant-based chemical EOR, the economics and the feasibility of the chemical EOR operation are defined by surfactant retention. In carbonates, the surfactant retention is very high and it lies in the range of 0.4–1.6 mg/g of rock for anionic surfactants, which is unfavorable to surfactant-polymer-based enhanced oil recovery activities^2,17,18^. The retention of surfactant is high at salinities associated with Type 2 microemulsion. However, at these high salinities, the formed microemulsion with the surfactant slug would be trapped in the rock pores. This adsorbed oil can be remobilized by displacing it with a solution that has low salinity (typically, 75% of the optimum salinity) and it will reverse the phase behavior. Thus, to increase the recovery after the reversal of phase behavior. The injection of the polymer after the surfactant slug will decrease the viscosity of the chemical flood, enhancing oil displacement and its recovery. However, the propagation of the polymer front is an important parameter controlling the displacement during polymer drive. Thus, a delayed front will consequently defer the arrival of the expected oil bank, this delay will affect the economics of the chemical EOR project^11,19^. It is important to mention that the polymer front propagation is affected by two main factors. These factors include polymer acceleration and polymer retention. These two factors effectively delay/slows down the front. The deceleration of the polymer occurs due to the mechanical entrapment of polymer molecules and their adsorption onto the surface of rock^20,21^. Moreover, if the retention of polymer is high it will substantially reduce the displacement, and the recovery of the oil^8^.

The modeling of surfactant polymer flooding and prediction of its performance in carbonates is complex and difficult because dissolution behaviors and surface charges for carbonate vary due to the presence of metal oxides. It is mentioned by Kosmulski^22^ that these metal oxides have a higher point of zero surface charge as opposed to carbonates. Moreover, it is found by Stumm and Morgan^23^ that at a specific pH value, surface charge and oxide surface charge in carbonates are opposite in sign. This finding proves that during surfactant polymer flooding, the surface potential would change with time and space^24,25^. Thus, the ionic composition of surfactant polymer flood will differ from the injected flood, when it comes in contact with formation because of various geochemical reactions that will take place instantaneously. These geochemical reactions will lead to the retention of surfactant in the porous media^26,27^. Moreover, surfactant retention can be decreased by increasing the pH of the solution^28^. The chemicals used to increase pH are Na_2_CO_3_ and NaOH. The use of Na_2_CO_3_ will cause the dissolution of anhydrite and metal oxides, and scale formation^29^. Recently, it is suggested by Tagavifar et al*.*^18^, Wang et al*.*^30^ and Wang^31^ to use NaOH, and they mentioned that the use of NaOH in carbonates is anodyne because it does not react with calcite, dolomite, anhydrite, and quartz. Moreover, NaOH injection even at low concentrations is effective, as it will increase the pH of the solution to 12 while diminishing the dissolution of calcite even at high pH values^32^. Therefore, a comprehensive modeling approach is needed to capture the effects of rock mineralogy as well as fluid composition on surfactant retention during surfactant polymer flooding.

Thus, it is important to investigate the interaction (compatibility) of surfactant and polymer with formation fluids and rock because during chemical flooding they would mix through the process of dispersion and diffusion. Based on the literature above, one can note that the effect of polymer composition on surfactant retention with reservoir mineral constituents is still unclear due to the variable subsurface conditions such as ionic strength, fluid and rock compositions, and pH. Therefore, the objective of this study is to better estimate surfactant retention dynamics on a carbonate rock with heterogeneous polymer composition with certain surface properties. We utilized experimental data from the literature and developed a chemical-based surface complexation geochemical model. Moreover, it is important to mention that one cannot use hypothetical data of polymer composition and must rely on the real/measured composition. Therefore, we used the experimental calculated composition of polymer drive used by Wang et al*.*^30^ to analyze the effect of surfactant retention. The developed model is validated against different experimental works. The geochemical interactions between various chemicals (surfactant and polymer), oil, formation brine, and carbonate rock surfaces were explored. It is important to mention that in this study, we only considered the retention of surfactants with polymer drive. Thus, to characterize the carbonate surface, the in-situ effluent ionic composition of water along with reservoir rock compositions. We simulated this complex geochemical process with the inverse modeling approach and combined it with the kinetic dissolution of solid species in the Phreeqc (Geochemical speciation software developed by Parkhurst and Appelo^33^). We utilized the surface complexation modeling approach to predict the surface speciation as a function of polymer drive composition to estimate the retention of surfactant on the reservoir rock surface.

## Surface complexation-based geochemical model

The activity of surfactant and their transport in carbonates is controlled by their adsorption/desorption. To determine the influence of polymer drive composition to recover the adsorbed surfactants, various techniques can be used. These techniques include an empirical approach and a modeling approach. The empirical approaches such as Langmuir isotherm equations, and Freundlich isotherm equations uses the distribution coefficients. However, these empirical approaches ignore the variation in surface charge with the change in the composition of injected fluid, ionic strength and pH of reservoir fluid, that control surfactant retention, and related geochemical reactions^18^. Thus, to determine the activity of surfactants, the utilization of sophisticated geochemical models (surface complexation models) is suggested as a comprehensive approach to overcome the limitations of adsorption isotherm models. It is imperative to mention that the various physicochemical mechanisms of the proposed geochemical models are included by certain geochemical reactions where the ionic species adsorbs on the surface of the rock-forming various functional groups. Moreover, during surfactant-polymer flooding, a unique surface charge and surface potential will develop between the surface of the reservoir and the chemical flood solution. Thus the modification of rock surface charge and surface potential variation will modify the electrical double layer (EDL) thickness that is used to estimate the characteristics of ionic species around a charged rock surface^34^. After estimating the rock surface charge and its surface potential, the thickness of the water film is estimated by employing the DLVO theory (where DLVO stands for Dejaguin, Landau, Verwey, and Overbeek). This theory describes the various interactions (forces of attraction and repulsion) around a charged rock surface during surfactant polymer flooding. The DLVO theory states that stable film water will form between oil and rock surfaces due to competing intermolecular surface forces, named the disjoining pressure^35^. This disjoining pressure is the summation of electrostatic forces, van der Waals forces, and structural forces^26,36–38^. The estimation of these forces requires the effect of variation in surface charge and surface potential during chemical flooding. Thus, the effect of surfactant retention can be modeled with chemical models such as surface complexation models. These models consider different geochemical reactions, mass and charge balance, intrinsic stability constants, the geochemical composition of brine, oil, solid, and surface species and solutions, surface sites, and surfactant retention on the mineral surface sites. In this research study, we used the diffuse layer surface complexation model because this model has the ability to simulate mineral surfaces, various sites, and the retention of surfactants. Moreover, this model states that two layers of ions are parallel and they exist around the rock surface known as the stern layer and diffuse layer.

The different surface complexation reactions used for modeling the retention of surfactant on the surface of calcite and hydrous ferric oxides (HFO) are depicted in Table 1. The log-intrinsic stability constants in this table were empirically estimated by Ma et al*.*^39^, and ShamsiJazeyi et al*.*^40^ Moreover, it is assumed in this study that the surfactant is an anionic specie with a molecular weight of 452 g/mole, denoted by A^−^. The detailed composition of carbonate rock, its surface area, type of different sites, and density are provided in Table 2. It is evident from the rock composition given in Table 2 that calcite is the major mineral with 99.19 wt. % and hydrous ferric oxide (HFO) is the trace mineral with just 0.81 wt. %. The various surface complexation reactions depicting the surfactant retention on the surfaces of calcite and HFO with their respective log-intrinsic stability constants are provided in Table 1.

**Table 1:** Surface complexation reactions used with intrinsic stability constants.

Nos.	Surface complexation reactions	Intrinsic stability constant (Log K_int_)			
		Tagavifar et al.^18^	Temperature corrected model		
		78 °C	60 °C	80 °C	100 °C
**Calcite-brine interface**					
CB1	>CO_3_H ↔ >CO_3_^−^ + H^+^_(aq)_	− 6.20	− 5.90	− 6.20	− 6.60
CB2	>CO_3_H + Ca^2+^_(aq)_ ↔ >CO_3_Ca^+^ + H^+^_(aq)_	− 6.10	− 5.80	− 6.20	− 6.50
CB3	>CaOH_2_ ↔ >aOH + H^+^_(aq)_	− 11.00	− 10.50	− 11.00	− 11.60
CB4	>CaOH ↔ >CaO^−^ + H^+^_(aq)_	− 14.00	− 13.52	− 14.00	− 14.64
CB5	>CaOH + H^+^ ↔ >CaOH_2_^+^	7.00	7.30	6.90	6.50
CB6	>CaOH + CO_3_^2−^ + H^+^_(aq)_ ↔ >CaCO_3_^−^ + H_2_O	− 2.60	− 2.50	− 2.60	− 2.80
CB7	>CaOH + CO_3_^2−^ + 2H^+^_(aq)_ ↔ > CaHCO_3_ + H_2_O	6.50	6.80	6.43	6.10
**Trace minerals–brine interface**					
TM1	>Fe_(w)_OH + H^+^ ↔ Fe_(w)_OH_2_^+^	8.49	8.82	8.46	8.10
TM2	>Fe_(w)_OH ↔ >Fe_(w)_O^−^ + H^+^	− 9.10	− 8.60	− 9.15	− 9.70
TM3	>Fe_(s)_OH + H^+^ ↔ >Fe_(s)_OH_2_^+^	8.49	8.90	8.44	7.90
TM4	>Fe_(s)_OH ↔ >Fe_(s)_O^−^ + H_2_^+^	− 8.93	− 8.42	− 8.98	− 9.54
TM5	>Fe_(w)_OH + Fe^2+^ ↔ >Fe_(w)_OFeOH + 2H^+^	− 2.98	− 2.80	− 3.00	− 3.20
TM6	>Fe_(w)_OH + Fe^2+^ + H_2_O ↔ >Fe_(w)_OFe^+^ + H^+^	− 15.98	− 15.30	− 16.06	− 16.90
TM7	>Fe_(s)_OH + Fe^2+^ ↔ >Fe_(s)_OFe^+^ + H^+^	− 0.98	− 0.90	− 0.99	− 1.10
TM8	>Fe_(w)_OH + Ca^2+^ ↔ >Fe_(w)_OCa^+^ + H^+^	− 6.05	− 5.70	− 6.08	− 6.40
TM9	>Fe_(w)_OH + Mg^2+^ ↔ >Fe_(w)_OMg^+^ + H^+^	− 6.80	− 6.40	− 6.80	− 7.20
**Surfactant-minerals interface**					
SM1	>CO_3_H + A^−^_(aq)_ ↔ > CO_3_HA^−^_(aq)_	4.90	5.20	4.80	4.50
SM2	>CaOH + A^−^_(aq)_ ↔ >CaOHA^−^	0.70	1.01	0.65	0.29
SM3	>CaOH_2_ + A^−^_(aq)_ ↔ >CaOH_2_A	2.40	2.71	2.35	1.99
SM4	>CO_3_Ca^+^ + A^−^_(aq)_ ↔ >CO_3_CaA	4.20	4.51	4.15	3.79
SM5	>Fe_(w)_OH + A^−^_(aq)_ ↔ >Fe_(w)_OHA^−^	3.90	4.21	3.85	3.49
SM6	>Fe_(w)_OFe^2+^ + A^−^_(aq)_ ↔ >Fe_(w)_OFeA	0.20	0.37	0.20	0.04
SM7	>Fe_(s)_OFe^+^ + A^−^_(aq)_ ↔ >Fe_(s)_OFeA	0.20	0.37	0.20	0.04

**Table 2:** Composition, surface areas, type of surface types, and site densities used in the simulation.

Mineral	Initial amount (wt. %)	Surface area (m^2^/g)	Site type	Site density (μmol/m^2^)
Calcite	99.19	1.1	>CO_3_OH, >CaOH_2_^+^	3.323
Hydrous ferric oxide (HFO)	0.81	600	>Fe_(w)_OH, >Fe_(s)_OH	3.740

It is imperative to mention that we added the oil/brine and oil/surfactant surface complexation reactions to the developed geochemical model, and this approach made our model unique and permits us to estimate the retention of surfactants in the presence of oil. The oil surface area and site density are 0.8 m^2^/g and 1 site/nm^2^, respectively^41^. We also utilized the total oil acid number (TAN) and total base number (TBN) for the oil/brine interface to include the number of carboxylic acid groups and nitrogen base groups, respectively. Moreover, it is imperative to mention that at high pH, the rock surface charge will become more negative for crude oils with high TAN values due to the conversion of the acid component to the saline form of the molecule. Thus, we incorporated the various surface complexation reactions for oil/brine and oil/surfactant interfaces in this research and included the effect of TAN and TBN in the developed geochemical model as shown later in the paper. Moreover, it is important to mention that the various log-intrinsic stability values for different surface complexation reactions and their respective site densities vary over a certain range of temperatures that will affect the water film thickness and wettability alteration. Where the stability of this film is measured by the disjoining pressure as previously described. Therefore, the use of the surface complexation model has the capability to capture the reservoir wettability alteration^26,42–46^. We determined the amount of adsorption/desorption sites in the porous media by considering the rock surface area, rock mineralogical composition, and site density. Parkhurst and Appelo^33^ mentioned that the number of functional groups is associated with the site density (N_s_) in site/nm^2^, and it is given as:

1 $$\gamma =\frac{{10}^{18}{S}_{BET}{m}_{ads}{N}_{s}}{V{N}_{A}},$$

where γ (mol/l) is the reactive surfaces/functional groups, N_s_ (site/nm^2^) is the site density, S_BET_ (m^2^/g) is the BET surface area, (Where BET stands for Brunauer–Emmett–Teller), m_ads_ (g) is the adsorbent mass, V(L) is the volume of the solution, and N_A_ (6.02214076 × 10^23^/mol) is the Avogadro’s number. It is important to mention that under in-situ formation conditions, the different reservoir fluids have attained equilibrium with the reservoir rock and discrete ionic species are adsorbed onto the surface of the formation. The apparent equilibrium constant (K_a_) in surface complexation has two terms: (K_int_) the constant of intrinsic stability and electrostatic distribution. The constant of apparent equilibrium is defined by:

2 $${K}_{a}={K}_{int} exp\left(\frac{-F{Z}_{c}\psi }{RT}\right),$$

where *F* is the Faraday constant (96,490 C/mol), *Z*_*c*_ is the variation in surface charge due to surface complexes formation, and *ψ* is the surface potential (Volts). For surface complexation reaction, the constant intrinsic stability (K_int_) is determined using Eq. (3), and the stoichiometric coefficients for this equation are described in Eq. (4).

3 $${K}_{int}=\frac{(>Y)(Z)}{(>W)(X)}.$$

4 $$> {\text{W}} + {\text{X}} = \, > {\text{Y}} + {\text{Z}},$$

It is imperative to mention that usually the temperature of subsurface hydrocarbon reservoirs increases with the increase in depth and the temperature of a reservoir is typically more than 298.15 K. Thus, for aqueous reactions and dissolution/precipitation reactions, the equilibrium constant is determined by using the van’t Hoff equation that is as follows:

5 $$\frac{d[ln{K}_{i}\left(T\right)]}{dT}=\frac{\Delta {H}_{i}^{o}(T)}{R{T}^{2}},$$

where $\Delta {H}_{i}^{o}$ is a function of temperature and it is the standard heat of reaction for *i*th chemical reaction. Additionally, it is assumed that $\Delta {H}_{i}^{o}$ remains constant through a narrow temperature range and thus after integration the Eq. (5) yields the following equation:

6 $$ln\frac{{K}_{i}({T}_{2})}{{K}_{i}({T}_{1})}=\frac{\Delta {H}_{i}^{o}}{R}\left(\frac{1}{{T}_{1}}-\frac{1}{{T}_{2}}\right),$$

The Equation above (Eq. 6) is utilized to determine the equilibrium constant for a chemical reaction at any temperature T_2_ by utilizing the equilibrium constant value at temperature T_1_^46^. However, the value of $\Delta {H}_{i}^{o}$ is not reported for surface complexation reactions. Thus, we suggest using the following analytical equation to calculate the dependence of temperature for intrinsic stability constants^33^:

7 $${Log}_{10}K={A}_{1}+{A}_{2}T+\frac{{A}_{3}}{T}+{A}_{4}{Log}_{10}T+\frac{{A}_{5}}{{T}^{2}}+{A}_{6}{T}^{2}.$$

where T is the reservoir temperature in Kelvin (K) and A_1_ through A_6_ are geochemical parameters adjusted to match the equilibrium constants at 298.15 K. Then, these adjusted A_1_ through A_6_ parameters are utilized to determine the equilibrium constants at different temperatures at 60 °C, 80 °C, and 100 °C for calcite/brine, oil/brine, trace minerals/brine, surfactant/minerals, and oil/surfactant surface complexation reactions given in Tables 1, 6, 7, 10 and 11.

It is important to mention that the geochemical evaluation of polymer drive composition on surfactant retention in carbonates using the surface complexation modeling is performed with the Phreeqc geochemical simulator. The simulator first reads the thermodynamic data of the system provided in a database file such as llnl, Phreeqc, and Pitzer. Then the simulator processes the whole input data file. The program starts the simulation after considering any new thermodynamic data provided in the data file. In this research, we added the surface complexation reactions in the data file. The program then creates updated lists of elements, compounds, phases, and various species such as aqueous, exchange, and surface species. After executing the newly provided data, the program performs the following different calculations for a transport problem: (1) initial solution or speciation calculations, (2) initial exchange calculations, (3) initial surface calculations, (4) initial gas-phase calculations, (5) advective-transport calculations, (6) advective- dispersive transport calculations. The conservation of mass for a chemical species that is injected and transported in porous media yields the following reactive transport equation:

8 $$\frac{\partial C}{\partial t}=-v\frac{\partial C}{\partial x}+{D}_{L}\frac{{\partial }^{2}C}{{\partial x}^{2}}-\frac{\partial q}{\partial t}.$$

where *C* (mol/kgw) is chemical specie concentration in water, *t*(s) is time, *v*(m/s) is pore water flow velocity, *x*(m) is distance, *DL* (m^2^/s) is the hydrodynamic dispersion coefficient, and *q* (mol/kgw in the pores) is concentration in the solid phase. The first term on the right-hand side ($-v\frac{\partial C}{\partial x}$) represents advective transport, second term (${D}_{L}\frac{{\partial }^{2}C}{{\partial x}^{2}}$) represents dispersive transport, and $-\frac{\partial q}{\partial t}$ is the change in concentration in the solid phase due to geochemical reactions.

The transport part of the above Eq. (8) is solved with an explicit finite difference scheme that is forward in time, central in space for dispersion, and upwind for advective transport. The chemical interaction term for each element is calculated separately from the transport part for each time-step and is the sum of all equilibrium and non-equilibrium reaction rates. The numerical approach follows the basic components of the reactive transport equation in a split-operator scheme^33^. With each time step, first advective transport is calculated, then all equilibrium and kinetically controlled chemical reactions, thereafter dispersive transport, which is followed again by calculation of all equilibrium and kinetically controlled chemical reactions. The scheme differs from the majority of other hydrogeochemical transport models (Parkhurst and Appelo^33^) in that kinetic and equilibrium chemical reactions are calculated both after the advection step and after the dispersion step. This reduces numerical dispersion and the need to iterate between chemistry and transport.

## Results and discussion

The maximum amount of oil from carbonate reservoirs can be recovered with the CO_2_-based EOR under supercritical conditions^47,48^. However, the injection of CO_2_ could lead to the precipitation/deposition of asphaltene^49–51^, dissolution of carbonate particles and cementation of asphaltene and dissoluted particles^16^ and sludge formation^52^. Thus, an integrated geochemical model is developed to determine the effect of polymer drive on surfactant retention as described in the preceding section of this study. This section of the paper includes the validation of the suggested surfactant-polymer-based surface complexation model against single and two-phase experiments from the literature. Additionally, we determined the effects of various parameters on surfactant retention. These parameters include the composition of the polymer drive, its temperature, salinity, concentration of hard ions in the polymer drive, and HFO and Mg surface complexation reactions.

### Validation of geochemical model

The development of a geochemical model using the surface complexation approach is a complex and difficult process as it considers the properties of reservoir rock, formation and injected water composition, various involved geochemical reactions, system thermodynamic conditions, and injected fluid flow rate^53,54^. Thus, it is necessary to confirm the validation of the developed geochemical model with the experimental data. This section of the paper consists of geochemical model validation against single and two-phase experimental data. We used surfactant effluent concentration and solution pH data to validate the developed model, and captured surfactant retention, with polymer drive during the SP floods. It is important to mention that the adsorption of the polymer was not considered in this study because it is out of the scope of this work. A detailed explanation of the geochemical model validation with the experimental datasets is presented below.

#### SP study of Tagavifar et al.^18^

To present the ability of the developed geochemical-based surface complexation model to determine the insights into the various factors during polymer drive on the retention of surfactant. We selected an experimental and modeling study of surfactant retention. In this part of the paper, the modeling results of the developed geochemical model are compared with the experimental and modeling work performed by Tagavifar et al.^18^. We selected this study because they injected anionic surfactant in carbonate rock and tested surfactant-polymer flooding at a single phase. The composition of the carbonate sample used in the geochemical modeling is previously depicted in Table 2. Detailed information about the properties of carbonate and modeling input are presented in Table 3.

**Table 3:** Reservoir rock properties used for simulation (Tagavifar et al*.*^18^).

Parameter	Value
Core porosity (%)	18.5
Core length (cm)	30.48
Temperature (°C)	78
Lithology (%)	Calcite (99.19), Iron as HFO (0.81)
Elements or pseudo-elements	Aluminum, calcium, chloride, iron, magnesium, sodium, sulfate
Solid species	Calcite, Fe-dolomite, and anhydrite
Number of gridblocks	20 × 1 × 1 (1D model)
Shifts	8 (surfactant slug), 40 (polymer drive)
Timestep (s)	4320
Diffusion coefficient (m^2^/s)	0.0
Dispersivity (m)	0.02
Flow direction	Forward
Boundary conditions	Flux–flux

**Figure 2:**
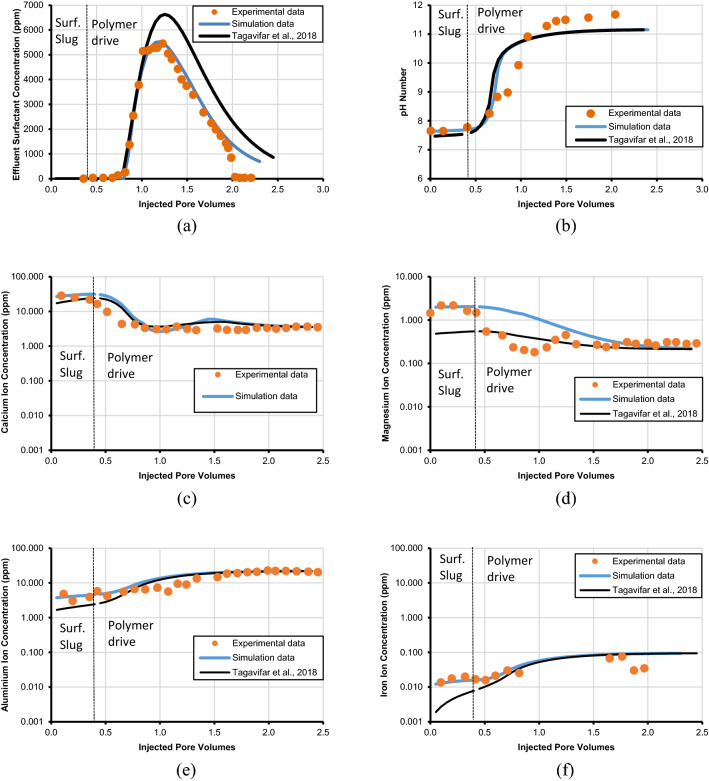
Comparison of the developed model with the experimental and simulation data of Tagavifar et al.^18^; (**a**) effluent surfactant concentration, (**b**) pH, (**c**) calcium concentration, (**d**) magnesium concentration, (**e**) aluminum concentration, (**f**) iron concentration.

The concentration of different ionic species and charge balance for the formation brine, surfactant slug, and polymer drive are shown in Table 4. The concentration of total dissolved solids (TDS) in formation brine is 65,000 ppm, surfactant slug, and polymer drive are 61,874 and 30,974 ppm, respectively. It is important to mention that we used C_24_–PO_45_–EO_3_0–COONa (0.65 wt%) in the surfactant slug and Flopaam 3330S hydrolyzed polyacrylamide (HPAM) (0.35 wt%) in the polymer drive. The different surface complexation reactions used in modeling include the reactions at the calcite/brine interface, HFO/brine interface, and the surfactant/mineral/brine interface are shown in Table 1. Additionally, in modeling, 0.4 pore volume of surfactant slug is injected that is followed by 2.0 pore volume of polymer drive at a temperature of 78 °C. The results are compared against both experimental and simulation data as depicted in Fig. 2 for the pH, concentration of surfactant in the effluent, and concentrations of ionic species in the effluent including aluminum, calcium, iron, and magnesium ions. It is evident from the results presented in Fig. 2(a) through 2(f) that our results are in good match with the effluent experimental data of (a) surfactant, (b) pH, (c) calcium, (d) magnesium, (e) aluminum, and (f) iron effluent concentrations as opposed to the modeling results of Tagavifar et al.^18^.

**Table 4:** Compositions of different waters used in the simulation runs (Tagavifar et al*.*^18^).

Salinity unit	ppm								Ppm	meq/ml	
Ions	Ca^2+^	Na^+^	Cl^−^	SO_42_^−^	Mg^2+^	Al^3+^	Fe^2+^	A^−^	TDS	Anions	Cations
Brine composition	0	25,569.22	39,430.78	0	0	0	0	0	65,000	1.1	1.1
Surfactant slug composition	0	23,992.25	24,356.09	13,525.99	0	0	0	15,000	61,874.33	1.0	1.0
Polymer drive composition	0	11,837.04	5611.3	13,525.99	0	0	0	0	30,974.33	0.5	0.5

It is important to mention that this study obtained a better match than the modeling results of Tagavifar et al.^18^ due to three approaches that are introduced by this study. The first approach introduced by this study is to use a brine composition that is in equilibrium with the formation instead of using synthetic brine composition that is not in equilibrium with the formation (Table 4). The composition of equilibrium brine used in this study and synthetic brine used by Tagavifar et al.^18^ are shown in Table 5. We suggested the use of equilibrium brine because Tagavifar et al.^18^ allowed the brine to remain in the core overnight but used synthetic brine in modeling, which resulted in discrepancy. Thus, when injected water comes in contact with the formation, certain geochemical reactions will occur and then equilibrium will be achieved. The different geochemical reactions that would occur include aqueous reactions, dissolution/precipitation reactions, ion-exchange reactions, and surface complexation reactions. It is evident from the composition of equilibrium and synthetic brine presented in Table 5 that they have the same amount of sodium and chloride ions. However, the amount of aluminum, calcium, iron, and magnesium ions concentration has been increased from naught to 2, 16, 0.01, and 1.45 ppm, respectively. It is imperative to mention that the water composition used by Tagavifar et al.^18^ and Wang^31^ lack magnesium and iron ions. We performed a detailed batch reaction modeling and found that these species will instigate after attaining equilibrium with the carbonate core.

**Table 5:** Compositions of equilibrium brine used in this work versus synthetic brine used by Tagavifar et al.^18^.

Salinity unit	ppm							
Ions	Ca^2+^	Na^+^	Cl^−^	SO_4_^2-^	Mg^2+^	Al^3+^	Fe^2+^	Total Salinity
Synthetic brine composition (Tagavifar et al.^18^)	0	25,569.22	39,430.78	0	0	0	0	65,000
Equilibrium brine composition	16	25,569.22	39,430.78	0	1.45	2	0.01	65,019.46

**Figure 3:**
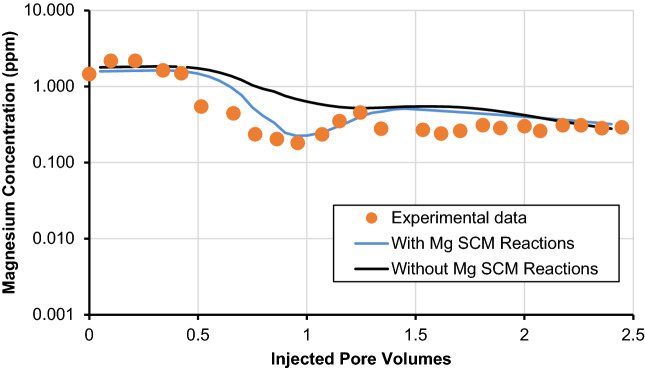
Effect of magnesium ion concentration in effluent and comparison of with and without the usage of magnesium surface complexation reactions.

The second approach that we employ is to slightly tune the log-intrinsic stability constant (K_int_) for the various surface complexation reactions along with the rock/brine/oil/surfactant interface. The tuned values of log-intrinsic stability constants for which we got the match are presented in Table 6. It is important to mention that this technique of tuning K_int_ has been utilized by several researchers^43,44,46^. The third approach that we utilized is that we added two magnesium surface complexation reactions; one for calcite/brine and the other for calcite/surfactant interface surface complexation reaction. The reason for adding these equations is that the effluent concentration of magnesium ion was not properly matched between 0.4 and 1.25 pore volumes as shown in Fig. 2d. We used the inversion analysis technique to determine the K_int_ values. The various calculated K_int_ are provided in Table 7. It is imperative to mention that these three approaches assisted us to obtain an improved match with the experimental data, the comparison is shown in Fig. 3.

**Table 6:** Tuned log-intrinsic stability constants for surfactant-minerals interface reactions.

Nos.	Surfactant-minerals interface reaction	Log-intrinsic stability constant (K_int_)				
		Tagavifar et al.^18^	Tuned model	Temperature corrected model		
		78 °C	78 °C	60 °C	80 °C	100 °C
SM1	>CO_3_H + A^−^_(aq)_ ↔ >CO_3_HA^−^_(aq)_	4.90	4.90	5.20	4.80	4.50
SM2	>CaOH + A^−^_(aq)_ ↔ >CaOHA^−^	0.70	0.20	0.37	0.20	0.04
SM3	> CaOH_2_ + A^−^_(aq)_ ↔ > CaOH_2_A	2.40	2.00	2.31	1.95	1.59
SM4	>CO_3_Ca^+^ + A^−^_(aq)_ ↔ >CO_3_CaA	4.20	4.00	4.31	3.95	3.59
SM5	>Fe_(w)_OH + A^−^_(aq)_ ↔ >Fe_(w)_OHA^−^	3.90	5.90	6.21	5.85	5.49
SM6	>Fe_(w)_OFe^2+^ + A^−^_(aq)_ ↔ >Fe_(w)_OFeA	0.20	0.20	0.37	0.20	0.04
SM7	>Fe_(s)_OFe^+^ + A^−^_(aq)_ ↔ >Fe_(s)_OFeA	0.20	0.20	0.37	0.20	0.04

**Table 7:** Magnesium surface complexation reactions added for better experimental data match.

Nos.	Surfactant-minerals interface reaction	Log-intrinsic stability constant (K_int_)			
		Developed model	Temperature corrected model		
		78 °C	60 °C	80 °C	100 °C
CB7	> CO_3_^−^ + Mg^2+^ ↔ > CO_3_Mg^+^	− 6.65	− 6.30	− 6.68	− 7.07
SM8	> CO_3_Mg^+^ + A^−^_(aq)_ ↔ > CO_3_MgA	5.70	6.03	5.67	5.31

#### SP study of Wang^31^

We validated the developed geochemical surface complexation model with a surfactant-polymer coreflooding experiment performed by Wang^31^. They performed a number of single and two-phase dynamic surfactant retention experiments. As we validated the developed geochemical model in the section above with Tagavifar et al.^18^. Thus, we used the two-phase experiment names A-5 performed by Wang^31^ to determine surfactant retention. It is imperative to mention that Wang^31^ in the two-phase experiment utilized Flopaam 3330S hydrolyzed polyacrylamide (HPAM) and kept its concentration at 0.35 wt% in the polymer drive. The mixture of surfactant composed of C_24_–PO_45_–EO_3_0–COONa and its concentration was kept at 0.65 wt%. Additionally, the core that was used to determine surfactant retention at two-phase was Indiana Limestone composed of calcite 97.7%, quartz 0.9%, K-feldspar 0.6%, illite, and mica 0.6%, and Fe-dolomite 0.2%. The experimental details of surfactant retention are available elsewhere (Wang^31^), where they injected 2 pore volumes of polymer drive after 0.4 pore volume of surfactant slug. The compositions of brine, SP slug, and polymer drive are listed in Table 8. Similar conditions of the single-phase experiment were honored for this coreflood where surfactant concentration was 0.65 wt%.

**Table 8:** Compositions of different waters used for two-phase dynamic surfactant retention experiment (A-5) of Wang^31^.

Salinity unit	ppm	meq/ml							
Ions	Ca^2+^	Na^+^	Cl^−^	SO_4_^2−^	Mg^2+^	OH^−^	TDS	Anions	Cations
Brine composition	0	25,188	36,112	3700	0	0	65,000	1.10	1.10
Surfactant slug composition	0	25,005	33,170	3700	0	1276	63,150	1.0	1.0
Polymer drive composition	0	12,967	14,607	3700	0	1276	32,550	0.5	0.5

**Figure 4:**
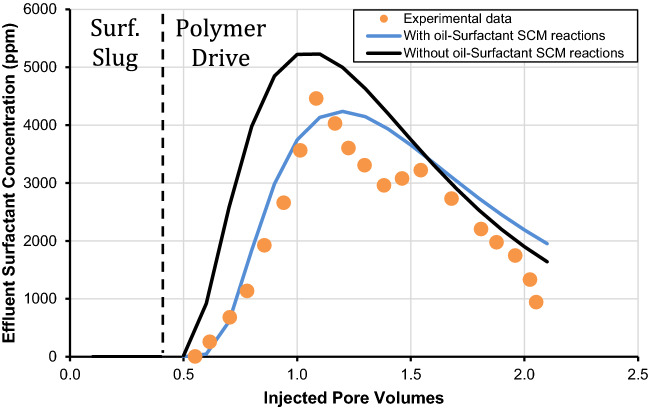
Effect of effluent concentration of surfactant and comparison of with and without the usage of oil-surfactant surface complexation reactions.

**Figure 5:**
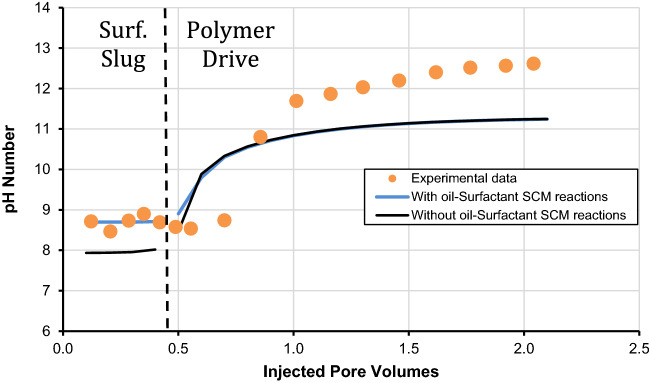
Effect of pH and comparison of with and without the usage of oil-surfactant surface complexation reactions.

It is important to mention that Wang^31^ performed the A-5 experiment to calculate the recovery of oil recovery and retention of surfactant in carbonates by measuring the pH and the concentration of surfactant in the effluent surfactant. Thus, we performed history matching of the laboratory data with the developed geochemical-based surface complexation model. It is imperative to mention that this is the first study that uses oil/surfactant surface complexation reactions to analyze the effect of polymer drive composition on surfactant retention. We used the values of K_int_ for surface complexation reactions along with the oil/brine interface. The K_int_ values for these reactions are listed in Table 9 as provided by Brady and Thyne^55^. The oil/surfactant surface complexation reactions and their respective K_int_ values were determined by inversion analysis as shown in Table 10. The values of K_int_ for a two-phase system have been followed recently by several researchers^2^. It is worthy to mention that with the use of various magnesium reactions, oil/brine, and oil/surfactant surface complexation reactions, we got an excellent history match for pH and surfactant concentration in the effluent as presented in Figs. 4 and 5, respectively. The significance of various oil/surfactant surface complexation reactions is emphasized in the latter figures by matching them with oil/brine interface reactions alone, where a reasonable history match is only possible when both (oil/brine, and oil/surfactant) surface complexation reactions were used. The history matching of two-phase surfactant retention coreflood affirms the validity of our developed geochemical surface complexation approach. Our results confirm that our developed geochemical approach can successfully estimate surfactant retention in the presence of the oil. Additionally, Wang^31^ reported that the retention of surfactant at 1.0 pore volume for the A-5 experiment was 0.17 mg/g and this study estimated that the surfactant retention is 0.15 mg/g. Thus the results are comparable to the amount of surfactant retention determined with the developed geochemical surface complexation model.

**Table 9:** Surface complexation reactions with intrinsic stability constants at the oil-brine interface.

Nos.	Surfactant-minerals interface reaction	Log-intrinsic stability constant (K_int_)				
		Brady and Thyne^55^	Temperature corrected model			
		25 °C	60 °C	78 °C	80 °C	100 °C
OB1	–NH + H^+^ ↔ –NH_2_^+^	− 6.00	− 6.80	− 7.10	− 7.10	− 7.60
OB2	–COOH ↔ –COO^−^ + H^+^	− 5.00	− 5.60	− 5.90	− 5.90	− 6.30
OB3	–COOH + Ca^2+^ ↔ –COOCa^+^ + H^+^	− 3.80	− 4.20	− 4.43	− 4.46	− 4.71
OB4	–COOH + Mg^2+^ ↔ –COOMg^+^ + H^+^	− 2.60	− 2.77	− 2.85	− 2.86	− 2.96

**Table 10:** Surface complexation reactions with intrinsic stability constants at the oil-surfactant interface.

Nos.	Surfactant-minerals interface reaction	Log-intrinsic stability constant (K_int_)			
		Developed model	Temperature corrected model		
		78 °C	60 °C	80 °C	100 °C
OS1	–NH + A^−^_(aq)_ ↔ –NHA^−^	2.50	2.60	2.49	2.39
OS2	–COOH + A^−^_(aq)_ ↔ –COOHA^−^	6.00	6.10	6.00	5.90
OS3	–COOH + Ca^2+^ + A^−^_(aq)_ ↔ –COOCaA + H^+^	10.00	10.10	9.99	9.87
OS4	–COOH + Mg^2+^ + A^−^_(aq)_ ↔ –COOMgA + H^+^	9.60	9.70	9.59	9.49

### Sensitivity analysis

In this section, the effect of various parameters is investigated on the concentration of surfactant in the effluent and its retention in porous media. We analyzed several parameters during chemical flooding including the composition of injected polymer drive, the temperature of injected polymer drive, salinity/dilution of polymer drive, and different surface complexation reactions with HFO and Mg. It is important to mention that we performed sensitivity analysis by considering two-phase (water and oil) and considered the various surface complexation equations for brine/mineral, mineral/surfactant, magnesium surface complexation reactions, oil/brine, and oil/surfactant presented in Tables 1, 6, 7, 9 and 10, respectively. We validated the latter reactions against the two surfactant-based experiments performed in carbonates by Tagavifar et al.^18^ and Wang^31^ which are presented in the validation section of this paper. Table 11 presents the composition of formation brine, injected surfactant slug, and polymer drive and Table 12 shows the parameters used to perform the sensitivity analysis.

**Table 11:** Compositions of different fluid compositions used in the simulation runs for the sensitivity analysis study.

Salinity unit	ppm										meq/ml	
Ions	Ca^2+^	Na^+^	Cl^−^	SO_4_^2−^	Mg^2+^	HCO_3_^−^	K^+^	A^−^	OH^−^	TDS	Anions	Cations
Brine composition	0	25,569.22	39,430.78	0	0	0	0	0	0	65,000	1.11	1.11
Surfactant slug composition	323	11,002	20,138	2479	1425	74	348	15,000	0	35,789	0.62	0.62
Polymer drive composition (SP-B1)	515	12,850	23,561	3275	1800	0	0	0	0	42,000	0.73	0.73
Polymer drive composition (SP-B2)	515	15,603	27,807	3275	1800	0	0	0	0	49,000	0.85	0.85
Polymer drive composition (ASP-B3)	0	14,886	10,313	13,526	0	0	0	0	1276	40,000	0.65	0.65
Polymer drive composition (ASP-B4)	0	11,315	3033	13,526	0	0	0	0	2126	30,000	0.49	0.49
Polymer drive ASP-B3 composition (base case)	0	14,886	10,313	13,526	0	0	0	0	1276	40,000	0.65	0.65
Polymer drive ASP-B3 composition (2-times diluted)	0	7443	5157	6763	0	0	0	0	638	20,000	0.32	0.32
Polymer drive ASP-B3 composition (10-times diluted)	0	1489	1031	1353	0	0	0	0	128	3,000	0.06	0.06

**Table 12:** Surfactant retention determined using the developed SCM for the two-phase sensitivity analysis study.

Nos.	Parameter	Polymer type	Temperature (°C)	Salinity (ppm)	HFO and Mg surface complexation reactions	Surfactant retention (mg/g of rock)
Case 1	Polymer type	SP-B1	80	42,000	HFO and Mg	0.38
		SP-B2	80	49,000	HFO and Mg	0.40
		ASP-B3	80	40,000	HFO and Mg	0.13
		ASP-B4	80	30,000	HFO and Mg	0.11
Case 2	Injected chemical flood temperature (°C)	ASP-B3	60	40,000	HFO and Mg	0.11
		ASP-B3	80	40,000	HFO and Mg	0.13
		ASP-B3	100	40,000	HFO and Mg	0.14
Case 3	The salinity of injected chemical flood (ppm)	ASP-B3	80	40,000	HFO and Mg	0.13
		ASP-B3	80	20,000	HFO and Mg	0.15
		ASP-B3	80	4000	HFO and Mg	0.31
Case 4	HFO and Mg surface complexation reactions	ASP-B3	80	40,000	HFO and Mg	0.13
		ASP-B3	80	40,000	Without HFO and Mg	0.08
		ASP-B3	80	40,000	HFO alone	0.14
		ASP-B3	80	40,000	Mg alone	0.08

### Injected polymer drive composition effect

**Figure 6:**
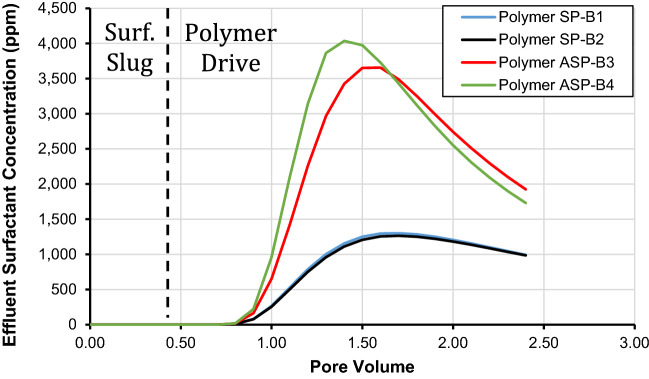
Effect of injected polymer type on effluent surfactant concentration using the developed SCM.

The influence of polymer drive composition during the chemical flood on surfactant effluent concentration is presented in Fig. 6. We used four different types of polymer drive compositions titled SP-B1, SP-B2, ASP-B3, and ASP-B4 as revealed/utilized by Wang et al.^30^. The ionic composition of different polymer drive compositions utilized in this study is provided in Table 11. It is important to mention that only injected polymer drive composition was changed while other parameters including chemical flood temperature, salinity, and surface complexation reactions were kept constant. Additionally, SP-B1 and SP-B2 have calcium, magnesium, and sulfate concentration of 515, 1,800, and 2479 ppm, respectively. However, ASP-B3 and ASP-B4 have zero concentration of calcium and magnesium, 13,526 for sulfate, and 1276 and 2126 ppm for hydroxyl ions in ASP-B3 and ASP-B4, respectively.

It is evident from Fig. 6 that the composition of the polymer during a chemical flood plays an important role in reducing the retention of surfactants in chemical flooding. It is imperative from this figure that the surfactant effluent concentration reaches 3974 and 3651 ppm after 1.5 pore volumes for the ASP-B3 and ASP-B4 polymer drive, respectively. However, for polymers SP-B1 and SP-B2, the concentration of surfactant in the effluent decreases to 1206 and 1250 ppm at 1.5 pore volumes. Moreover, regarding the retention of surfactant, Table 12 shows that SP-B1 and SP-B2 polymer drive results in 0.38 and 0.40 mg/g adsorption of surfactant, respectively. However, the retention of surfactant reduces to 0.13 and 0.11 mg/g for ASP-B3 and ASP-B4, respectively. Thus, our results confirm that the composition of polymer drive during chemical flooding plays a very important role in the retention of surfactants. Thus, the increase in surfactant effluent concentration is controlled by the composition of the polymer drive. The results indicate that for similar amounts and types of surfactants, a certain composition of polymer drive would produce masses of the surfactant. Based on this finding, it is worth mentioning that the geochemical composition of polymer drive in a chemical flood should be optimized in a way to decrease surfactant retention.

Moreover, it is imperative to mention the reason for high surfactant retention in SP-B1 and SP-B2, and low surfactant retention in ASP-B3 and ASP-B4. After detailed analysis, it is found that SP-B1 and SP-B2 have a significant concentration of hard ions (515 and 1800 ppm of calcium, and magnesium concentration, respectively) as shown in Table 11. Moreover, ASP-B3 and ASP-B4 have zero concentrations of hard ions. Thus based on these findings, it is interesting to highlight that presence of hard ions (calcium and magnesium) in polymer drive lead to an increase in the retention of surfactant. This is because the surfactants preferentially adsorb on the calcium and magnesium complexes at the rock surface which impedes the displacement of adsorbed components of carboxylic acid^56^. This is supported by the effluent surfactant concentration as presented in Fig. 6. Thus, the concentration of hard ions in polymer drive leads to a pronounced increase in surfactant retention and a decrease in its desorption. Additionally, the retention of surfactant in chemical flooding is a very case-dependent mechanism and it is controlled by reservoir rock composition and different impurities present in the rock, composition of crude oil, and reservoir thermodynamic conditions. Thus, it is vital to mention that findings cannot be generalized.

### Injected polymer drive temperature effect

This subsection presents injected polymer drive temperature effect on surfactant effluent concentration using the developed geochemical surface complexation model. During chemical flooding, the temperature of the flood is lower than the temperature of a reservoir. Thus, after the injection of polymer in the wellbore, the polymer solution moves down the wellbore through the tubings, and it would cool down the temperature of the tubing and the surroundings. We varied the temperature of the injected polymer between 60, 80 (base case), and 100 °C. It is imperative to note that the concentrations of different chemicals such as surfactants, solvents, cosolvents, flood salinity, and various surface complexation reaction were kept constant as listed in Table 12. We first injected a 0.4 pore volume of surfactant slug that was followed by 2.0 pore volumes of ASP-B3 polymer drive.

**Figure 7:**
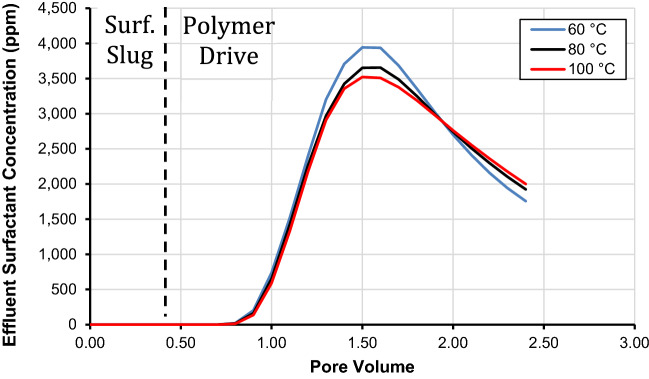
Effect of injected chemical flood temperature on effluent surfactant concentration using the developed SCM.

Figure 7 presents the influence of injected polymer drive temperature on the concentration of surfactant in the effluent. The results show that the influence of temperature on surfactant retention is significant. For the different injection temperatures of the injected polymer drive, one can note that when the temperature of the injected polymer drive is increased, the retention of surfactant increases. The analysis of surfactant concentration in the effluent is shown in Fig. 7, that during the chemical flood (surfactant slug and polymer flooding), the concentration of surfactant in the effluent at 1.5 pore volumes is 3940 ppm and 3650 ppm for 60 °C and 80 °C, respectively. However, the surfactant effluent concentration reduces to 3520 ppm for an injection temperature of 100 °C. Thus, it is evident from our results that the surfactant loss is more than 420 ppm in the reservoir at 100 °C as opposed to 60 °C. Additionally, the surfactant retention is 0.14 and 0.13 mg/g at 100 and 80 °C, respectively but it decreased to 0.11 mg/g at 60 °C as presented in Table 12. Therefore, our results depict that increasing the temperature of a chemical flood increases the retention of surfactant and decreases its desorption. The latter demonstrates that the surfactant retention is more of chemisorption as opposed to physio-adsorption. Thus, it is necessary to monitor the temperature of a chemical flood for a successful flooding operation. As it was previously mentioned that the influence of reservoir temperature on various geochemical reactions was captured through the Van’t Hoff equation for different aqueous and dissolution/precipitation reactions for their corresponding intrinsic stability constants.

### Injected polymer drive salinity effect

**Figure 8:**
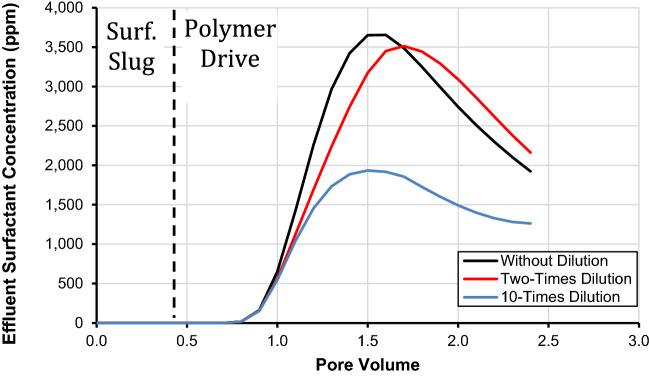
Effect of injected chemical flood salinity on effluent surfactant concentration using the developed SCM.

Figure 8 shows the influence of polymer drive salinity on surfactant retention and its concentration in the effluent that is characterized by the composition of polymer drive. We diluted the polymer drive two times and ten times and its salinity was kept at 20,000 and 4000 ppm, respectively, as presented in Table 11. In the base case of a polymer, drive salinity was kept at 40,000 ppm (black curve). It is important to mention that we altered only the salinity of the polymer drive while the values of the other parameters were kept constant.

It is apparent from Fig. 8 that decreasing the salinity of the polymer drive results in a decrease in surfactant retention. It is observed that for two-times diluted polymer drive and without dilution of polymer drive attained the values of 3078 and 3634 ppm at 1.5 pore volumes, respectively. Moreover, it is obvious from the results presented in this figure (Fig. 8) that the concentration of surfactant in the effluent reaches to1940 ppm after 1.5 pore volumes with ten-times diluted injected polymer drive. Thus, it is evident from our results that dilution of polymer drive is not effective in the early arrival of surfactant and is not helpful to increase its concentration in the effluent. Moreover, for the retention surfactant, as shown in Table 12, it is evident that the 10-times diluted polymer drive results in adsorption of 0.31 mg/g. However, if the polymer drive is diluted two-times and without its dilution, surfactant retention decreases to 0.15 and 0.13 mg/g, respectively. Hence, the retention of surfactant increases by decreasing the salinity of the polymer drive because of an increase in the force of repulsion between the mineral surface and adsorbed ionic species^57^. It is important to mention that the lowest level of surfactant retention was 0.13 mg/g, which was achieved through polymer drive without dilution. Therefore, a reduction in polymer drive salinity is not effective in increasing the recovery of a surfactant or decreasing the retention of surfactant. Thus a specific recipe for polymer drive could make the chemical flooding operation economical and cost-effective.

### HFO and Mg surface complexation reactions effect

**Figure 9:**
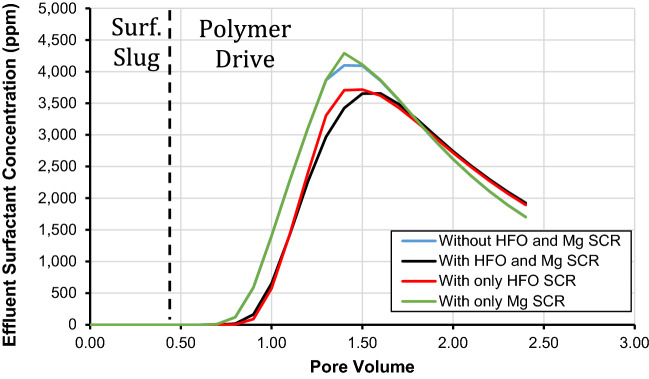
Effects of HFO and Mg surface complexation reactions on effluent surfactant concentration using the developed SCM.

The influence of including the hydrous ferric oxide and magnesium surface complexation reactions on simulating polymer driving during the chemical flood, and its effect on surfactant concentration in the effluent and, retention of surfactant is presented in Fig. 9 and Table 12, respectively. We considered four cases for this analysis that includes (i) HFO and Mg surface complexation reactions, (ii) without HFO and Mg surface complexation reactions, (iii) only HFO surface complexation reactions, and (iv) only Mg surface complexation reactions. The various surface complexation reactions utilized to develop the geochemical model are presented in Tables 1, 6, 7, 9 and 10. It is imperative to mention that the composition, temperature, and the salinity of polymer drive in the injected chemical flood were kept constant at ASP-B3, 80 °C, and 40,000 ppm, respectively.

The results presented for the base case where both HFO and Mg surface complexation reactions were considered resulted in 0.13 mg/g of surfactant retention. The latter level of surfactant retention is lower than the case where only HFO surface complexation reactions were considered (0.14 mg/g). Instead, the cases where both HFO and Mg surface complexation reactions were not considered and only Mg surface complexation reactions were considered resulted in surfactant retention that decreased to 0.082 and 0.081 mg/g, respectively as presented in Table 12. It is imperative to mention that the level of surfactant retention is consistent with surfactant concentration in the effluent as presented in Fig. 9. The figures presented that for the base case of both the HFO and Mg surface complexation reactions, the concentration of surfactant in the effluent is 3651 ppm at 1.5 pore volumes. However, for the case where we considered only HFO surface complexation reactions, the surfactant concentration in the effluent decreased to 3720 ppm at 1.5 pore volume. Furthermore, for the two cases, we considered both HFO and Mg surface complexation reactions and only Mg surface complexation reactions, the concentration of surfactant in the effluent increased to 4092 and 4110 ppm, respectively at 1.5 pore volume. Based on our findings, it is imperative to mention that during geochemical modeling of chemical flooding, we should consider the surface complexation reactions of all solid minerals found in the formation because if the existence of a mineral is ignored. It would result in erratic surfactant retention modeling and prediction. Similarly, the results present that the impact of HFO reactions on surfactant retention is more pronounced as opposed to surface complexation reactions for Mg. The latter highlights the significance of HFO active surface sites where the retention of surfactant is high due to their increased surface area as well as high ability to form hydrogen bonds^18^.

## Summary and conclusions

The objective of this study was to analyze the effect of polymer drive composition on surfactant retention during chemical flooding. We achieved the said objective by developing a novel geochemical model that captured mineral/brine/oil/surfactant/polymer interactions in carbonates. Our suggested approach assists in better forecasting the influence of polymer drive composition on surfactant retention, and the surfactant concentration and various aqueous species in the effluent. It is important to mention that this is the first study to test the effect of polymer composition on surfactant retention with the novel formulation of surface complexation modeling considering the effect of rock, brine, oil, surfactant, and polymer. Accordingly, four new surface complexation reactions with log-intrinsic stability constants have been proposed that honor the various interaction of oil, surfactant, and polymer interactions. The developed model is validated against including single-phase and two-phase coreflooding dynamic surfactant retention experimental studies reported in the literature. Furthermore, we examined the influence of different parameters of polymer drive on surface retention using the developed geochemical-based surface complexation model. The main outcomes of this study are summarized as follows:

- During chemical flooding specifically polymer drive, the oil-based surface complexation reactions should be considered to capture and estimate the surfactant retention.
- It is found that increasing the temperature of the polymer drive leads to an increase in surfactant retention, which concludes that the retention is a chemical process.
- Certain polymer drive compositions achieved the lowest surfactant retention levels (< 0.1 mg/g of rock) wherewith decreasing the salinity of the polymer drive is not an effective approach in reducing the surfactant retention. This happens due to an increase in the force of repulsion between the mineral surface and adsorbed ionic species.
- We found that there is an unfavorable effect of hard ions present in the polymer drive as the hard ions increase surfactant retention. This is due to the fact that hard ions preferentially adsorb at the rock surface.
- The results show that surfactant retention is more sensitive to HFO than Mg surface complexation reactions, which highlights the necessity of capturing the surface complexation reactions for rock-forming minerals to better model the chemical flooding and surfactant retention phenomenon.
- Surfactant retention during polymer drive and the related effect on oil recovery is very case-dependent and hence, the findings of this study cannot be generalized.

### Study limitations and future work

One of the limitations of this simulation study is that it ignores the change in flood salinity gradient and the corresponding impact on surfactant phase behavior and retention as a result of the polymer drive during chemical flooding. For future work, laboratory work will be conducted to further validate the observations from the literature and the numerical work on surfactant retention based on polymer drive composition. Furthermore, more sophisticated modeling approaches such as triple-layer surface complexation modeling can be used to better model surfactant retention.

## Data Availability

The datasets used and/or analyzed during the current study available from the corresponding author on reasonable request.
